# The Expansion of the *PRAME* Gene Family in Eutheria

**DOI:** 10.1371/journal.pone.0016867

**Published:** 2011-02-10

**Authors:** Ti-Cheng Chang, Yang Yang, Hiroshi Yasue, Arvind K. Bharti, Ernest F. Retzel, Wan-Sheng Liu

**Affiliations:** 1 Department of Dairy and Animal Science, The Center for Reproductive Biology and Health, College of Agricultural Sciences, The Pennsylvania State University, University Park, Pennsylvania, United States of America; 2 The Integrative Biosciences Program, Bioinformatics and Genomics Option, The Huck Institute of Life Sciences, The Pennsylvania State University, University Park, Pennsylvania, United States of America; 3 National Institute of Agrobiological Sciences, Tsukuba, Japan; 4 National Center for Genome Resources, Santa Fe, New Mexico, United States of America; University of Wyoming, United States of America

## Abstract

The *PRAME* gene family belongs to the group of cancer/testis genes whose expression is restricted primarily to the testis and a variety of cancers. The expansion of this gene family as a result of gene duplication has been observed in primates and rodents. We analyzed the *PRAME* gene family in Eutheria and discovered a novel Y-linked *PRAME* gene family in bovine, *PRAMEY*, which underwent amplification after a lineage-specific, autosome-to-Y transposition. Phylogenetic analyses revealed two major evolutionary clades. Clade I containing the amplified *PRAMEY*s and the unamplified autosomal homologs in cattle and other eutherians is under stronger functional constraints; whereas, Clade II containing the amplified autosomal *PRAME*s is under positive selection. Deep-sequencing analysis indicated that eight of the identified 16 *PRAMEY* loci are active transcriptionally. Compared to the bovine autosomal *PRAME* that is expressed predominantly in testis, the *PRAMEY* gene family is expressed exclusively in testis and is up-regulated during testicular maturation. Furthermore, the sense RNA of *PRAMEY* is expressed specifically whereas the antisense RNA is expressed predominantly in spermatids. This study revealed that the expansion of the *PRAME* family occurred in both autosomes and sex chromosomes in a lineage-dependent manner. Differential selection forces have shaped the evolution and function of the *PRAME* family. The positive selection observed on the autosomal *PRAMEs* (Clade II) may result in their functional diversification in immunity and reproduction. Conversely, selective constraints have operated on the expanded *PRAMEY*s to preserve their essential function in spermatogenesis.

## Introduction

Cancer/testis (CT) genes comprise a group of genes involved primarily in immunity and reproduction. They are expressed in various types of cancers when abnormally activated, whereas, the normal expression of CT genes is restricted mainly to the testis, but it has been detected also in other tissues such as fetal ovary [1]–[3]. CT genes have more than 240 members from 70 families. Twenty-four of these families are located on the human X-chromosome (CT-X) and two families, the *TSPY* (*testis-specific protein Y-linked*) gene family and the Y-linked *TPTE* (*transmembrane phosphatase with tensin homology*) pseudogene family, are on the Y-chromosome (Y-chr) [4]. Interestingly, many amplified CT gene families are located within direct or inverted repeats on the sex chromosomes (chrs) [1], [4]. The autosomal CT genes were conserved during evolution and play roles in spermatogenesis, fertilization, and apoptosis in malignant cells [5]–[7]. However, knowledge about the CT genes on the sex chrs is still limited. A comparative study suggested that the CT-X genes were subject to positive selection and evolved faster than the autosomal CT genes [4]. The Y-linked *TSPY* gene family is conserved among most mammalian species, and has 30–60 copies on the human Y-chr [8] and 50–200 copies on the bovine Y-chr (BTAY) [8], [9]. This family has a typical CT tissue-restricted expression pattern with functions in immunity and spermatogenesis [10]. In this study, we identified a novel Y-linked CT gene family, *preferentially expressed antigen in melanoma, Y-linked* (*PRAMEY*), and examined its evolution in Eutheria.

*PRAME*, as one of the CT genes, first identified as an antigen-encoding gene related to immunity in a melanoma cell line [2], is expressed predominantly in normal testis and melanoma, lung squamous cell carcinoma, and acute leukemia, and at much lower levels in the ovary and other tissues [2], [11]. The human *PRAME* gene, located on chromosome 22 (HSA22), encodes a protein with seven leucine-rich (LXXLL) motifs through which PRAME interferes with the retinoic acid receptor (RAR) pathway, and leads to the inhibition of RA-induced differentiation, growth arrest, and apoptosis [12]. Thus, PRAME functions as a transcriptional repressor in the signaling cascade, and the over-expression of *PRAME* results in tumorigenesis [12]. Similar to the other multi-copy CT genes, *PRAME* went through expansion and constituted a large gene family in most mammalian species [13], [14]. A previous phylogenetic analysis of the primate *PRAME* family has revealed that the expansion of the human paralogs is hominin-specific and occurred within the past three million years [13]. Several potential surface-accessible sites of the human PRAME protein have been identified under positive selection during evolution [13]. Even though the evolutionary pattern and oncogenic roles of the *PRAME* family have been studied in the human and rodent [2], [11]–[13], [15]–[17], the phylogeny of the *PRAME* orthologs in other mammalian species and the function of *PRAME* in normal tissues, such as testis, remain unclear.

To delineate the macro-evolution of *PRAME*, we analyzed the *PRAME* gene family in Eutheria. We discovered a bovine Y-linked *PRAME* family, namely *PRAMEY*, which was derived from an autosome-to-Y transposition and underwent amplification after the transposition. A phylogenetic analysis of *PRAME/PRAMEY* orthologs in Eutheria identified two major clades, which were subject to diverse selection pressures. The origination of the *PRAMEY* family and its unique expression patterns in spermatids suggest that it plays an important role in spermatogenesis.

## Results

### Discovery of the PRAMEY Family

**Figure 1:**
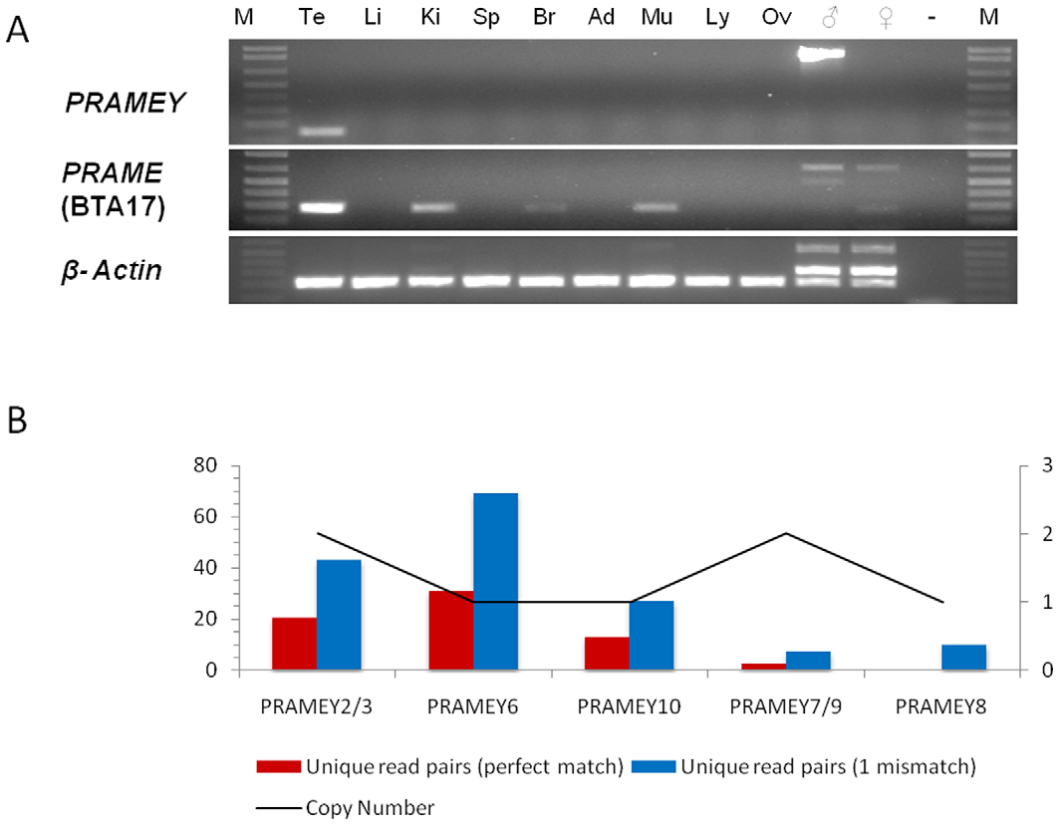
Expression patterns of *PRAME/PRAMEY* in cattle. **A.** RT-PCR results (lanes 2-10). *PRAMEY* is expressed specifically in the testis, whereas the autosomal *PRAME* is expressed in the testis (predominantly), kidney, brain and the muscle tissues. Bovine male genomic DNA-specific PCR (lanes 11–12) confirmed that *PRAMEY* is Y-specific. Te, testis; Li, liver; Ki, kidney; Sp, spleen; Br, brain (cerebrum); Ad, adrenal gland; Mu, muscle; Ly, lymph node; Ov, ovary; ♂, bovine male genomic DNA control; ♀, bovine female genomic DNA control; -, negative control (water); M, 1 kb DNA ladder. **B.** The expression of the *PRAMEY* loci by deep-sequencing analysis. The alignment of reads derived from deep-sequencing of selected cDNAs against coding regions of the *PRAMEY* loci (Table S2) reveals that seven of the 10 active *PRAMEY* genes are expressed differentially, six of which have significant numbers of both read-pairs matching exactly to the specific loci.

**Figure 2:**
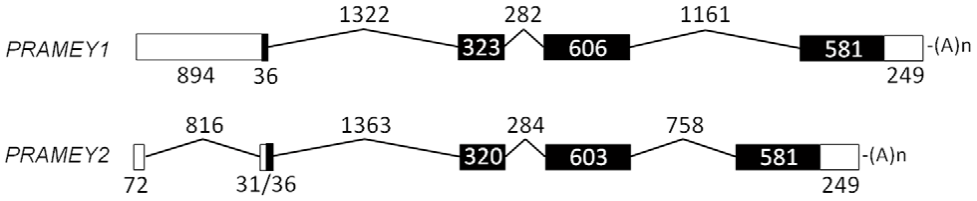
Genome structures of the bovine *PRAMEY* genes. Schematic representations of *PRAMEY1* and *PRAMEY2*. Compared to the *PRAMEY2* (GenBank acc. no. GU144302) that contains five exons, the first exon of the *PRAMEY1* (GenBank acc. no. GU144301) reads through to the second exon and forms a larger exon. The introns are drawn to scale. The open boxes represent UTR regions and the filled black boxes are coding segments (CDS). The numbers denote the length of exons, introns and CDS in bp. The polyA [(A)_n_] sites are indicated.

Two *PRAMEY* transcripts (*PRAMEY1* and *PRAMEY2*) were identified through a large-scale direct testis cDNA selection using a micro-dissected, PCR amplified BTAY probe. *PRAMEY1* is 99% identical to a predicted mRNA (GenBank acc. no. XM_001253165.1) located in a non-annotated bovine bacterial artificial chromosome (BAC) (GenBank acc. no. AC234911.1). This clone was validated as a Y-linked BAC by a male-specific PCR (Fig. 1). *PRAMEY2* is 99% identical to an mRNA (GenBank acc. no. NM_001077979) located in a bovine Y-BAC (GenBank acc. no. AC234853.4). Full-length mRNAs of both transcripts were obtained by RACE (rapid amplification of cDNA ends) (Fig. 2). The mRNA of *PRAMEY1* (GenBank acc. no. GU144301) is 2747 bp, with an open reading frame (ORF) from nucleotide (nt) 895 to 2436, and it encodes a peptide of 513 amino acids (aa). The mRNA of *PRAMEY2* (GenBank acc. no. GU144302) is shorter (1888 bp), with an ORF from nt 104 to 1639, encoding a peptide of 511 aa (Fig. 2). The similarity between the coding regions of *PRAMEY1* and *PRAMEY2* is 88% at the nucleotide level and 90% at the protein level.

To address the question whether more loci of *PRAMEY* are present on BTAY, we searched *PRAMEY1/2* against the bovine Y-BACs (available in NCBI) and identified a total of 10 potentially active *PRAMEY* paralogs (named *PRAMEY1-10*, Table S1) and 6 pseudogenes. The active- and pseudo-genes were mapped to a total of 11 Y-BACs, each containing one or two copies (Table S1). The pairwise similarity of the 10 active *PRAMEY* loci was >86%, with a 100% similarity between *PRAMEY2* and *PRAMEY3* in AC234853.4 (Table S2). *PRAMEY1* contains 4 exons whereas *PRAMEY2* contains 5 exons because the first exon of *PRAMEY1* reads through the second exon, resulting in a single, larger exon (Fig. 2). The first two introns in the coding regions are conserved across all the *PRAMEY* loci, with a slight difference in length (1289–1371 bp and 274–284 bp) (Fig. 2). A major difference is present in the last intron (Fig. 2): the size is 758 bp in *PRAMEY2/3/8*, compared to 1161–1212 bp in the remaining *PRAMEYs*. This difference is the result of an indel of 403–454 bp that is specific to BTAY.

The putative PRAMEY protein isoforms share an identity of ≥82%. Seven important leucine-rich motifs have been identified in the human PRAME protein [12]. The alignment of the bovine PRAMEY/PRAME with the human PRAME on HSA22 revealed that these motifs are highly conserved (Fig. S1).

In addition, we found a predicted gene (GenBank acc. no. XR_082974.1) located on BTA17. This gene shares ∼87% similarity with the identified Y-linked *PRAMEYs* (Table S2). Gene-specific PCR and sequencing (Table S3) confirmed the predicted *PRAME* on BTA17. This autosomal gene encodes a putative peptide of 410 aa and is located at 74.35 Mb close to two zinc-finger genes, *ZNF280A* (also known as *SUHW1*) and *ZNF280B* (*SUHW2*).

### Expression analysis of the bovine PRAMEY

Expression of the putative *PRAMEY* loci was investigated by deep-sequencing of the selected testis cDNAs using the Illumina GAIIx (see methods) and aligning the short sequence reads (pair-ends, 2×36 bp) against unique coding regions of the *PRAMEY* genes (Table S2). Seven of the 10 *PRAMEY* loci are active at the transcription level (*PRAMEY2/3/6-10*), and six of the seven loci have exactly matched read-pairs (Fig. 1B); in contrast, *PRAMEY1/4/5* have no matched reads. Further, *PRAMEY2/3*/*6* have more uniquely matched reads (>20), suggesting a higher expression level at these loci. Taken together with the RACE result, at least eight of the 10 loci on BTAY have been confirmed to be active at the transcription level.

**Figure 3:**
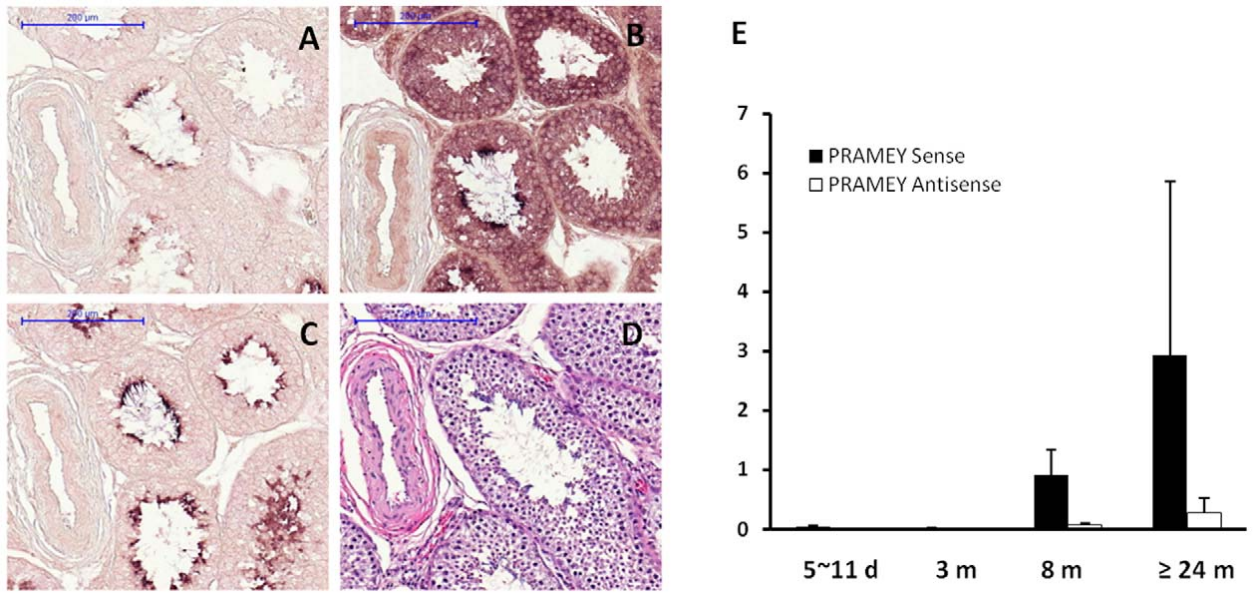
Spatial and temporal expression patterns of the sense and antisense RNA of the bovine *PRAMEY2* in adult bovine testis. **A.** The sense RNA of *PRAMEY2* is expressed specifically in spermatids. **B.** The antisense RNA of *PRAMEY2* is expressed broadly across seminiferous tubules with a predominant expression in spermatids. Sense and antisense RNAs of *PRAMEY2* were detected by DIG-labeled cRNA probes. **C.** The bovine *PRM1*gene was used as positive control, and there is no antisense mRNA of *PRM1* detected in the bovine testis [38]. **D.** Haematoxylin and Eosin (H&E) staining is shown. Scale: bar  = 200 µm. **E.** Temporal expression pattern of *PRAMEY2*. The relative expression levels of the *PRAMEY2* sense and antisense transcripts at different ages (X-axis), measured by the strand-specific qPCR, were normalized by the 18S rRNA (Y-axis). The *PRAMEY2* sense RNA is expressed very low in earlier stage, but up-regulated in the 8 months and 2 years-old testis. Similarly, antisense RNA of *PRAMEY2* is detected in the 8 months and 2 years-old testis. Values are means ± SD of the three biological replicates.

RT-PCR analysis (Table S3) across nine tissues revealed that *PRAMEY2* was expressed specifically in the testis. In contrast, the autosomal *PRAME* gene on BTA17 was expressed highly in the testis, and low in the kidney, brain and muscle (Fig. 1A). *In situ* hybridization (ISH) of *PRAMEY2* cRNA probes (Table S4) revealed that both sense and antisense transcripts of *PRAMEY2* were expressed in adult testis (Fig. 3). The sense RNA of *PRAMEY2* was expressed specifically in spermatids (Fig. 3A), whereas the antisense RNA was expressed in all cell types in the seminiferous tubules, with the highest expression occurring in spermatids (Fig. 3B). Quantitative (q) RT-PCR analysis of *PRAMEY2* indicated that the expression of the sense RNA was low in 5-11-day and 3-month-old testes, but up-regulated in 8-month- and 24-month-old testes (Fig. 3E); the expression of antisense *PRAMEY2* RNA increased slightly with age.

### Phylogenetic tree of the PRAME/PRAMEY family

To investigate the evolution of *PRAME/PRAMEY*, the sequences of multiple *PRAME* loci in the human, chimpanzee, orangutan, mouse, rat and cattle were retrieved from NCBI (Table S2) [18]. A single autosomal ortholog was found in dog and horse. Multiple *PRAME* loci were detected on the pig chr 6 (SSC6), similar to the expansions observed in primates (HSA1, PTR1 and MMUL1) and rodents (MMU4 and RNO5/14) [13]. Since SSC6 has not been well-annotated, the corresponding matched regions were collected and aligned with the HSA22 ortholog by Splign [19] to confirm gene structures and splicing signals/sites, which gave rise to 10 swine orthologs containing long ORFs (ranging from 470 to 528 aa) with corresponding splicing sites (Table S2). In addition to the autosomal copies, we found X-linked *PRAME* (*PRAMEX*) in rodents and horses. However, we did not identify any ortholog of *PRAME* in the non-eutherian lineages examined, including opossum, platypus, chicken, frog and zebrafish, all of which have a genome sequence coverage of ≥6X, implying that the *PRAME* gene family is present in eutherian mammals only.

**Figure 4:**
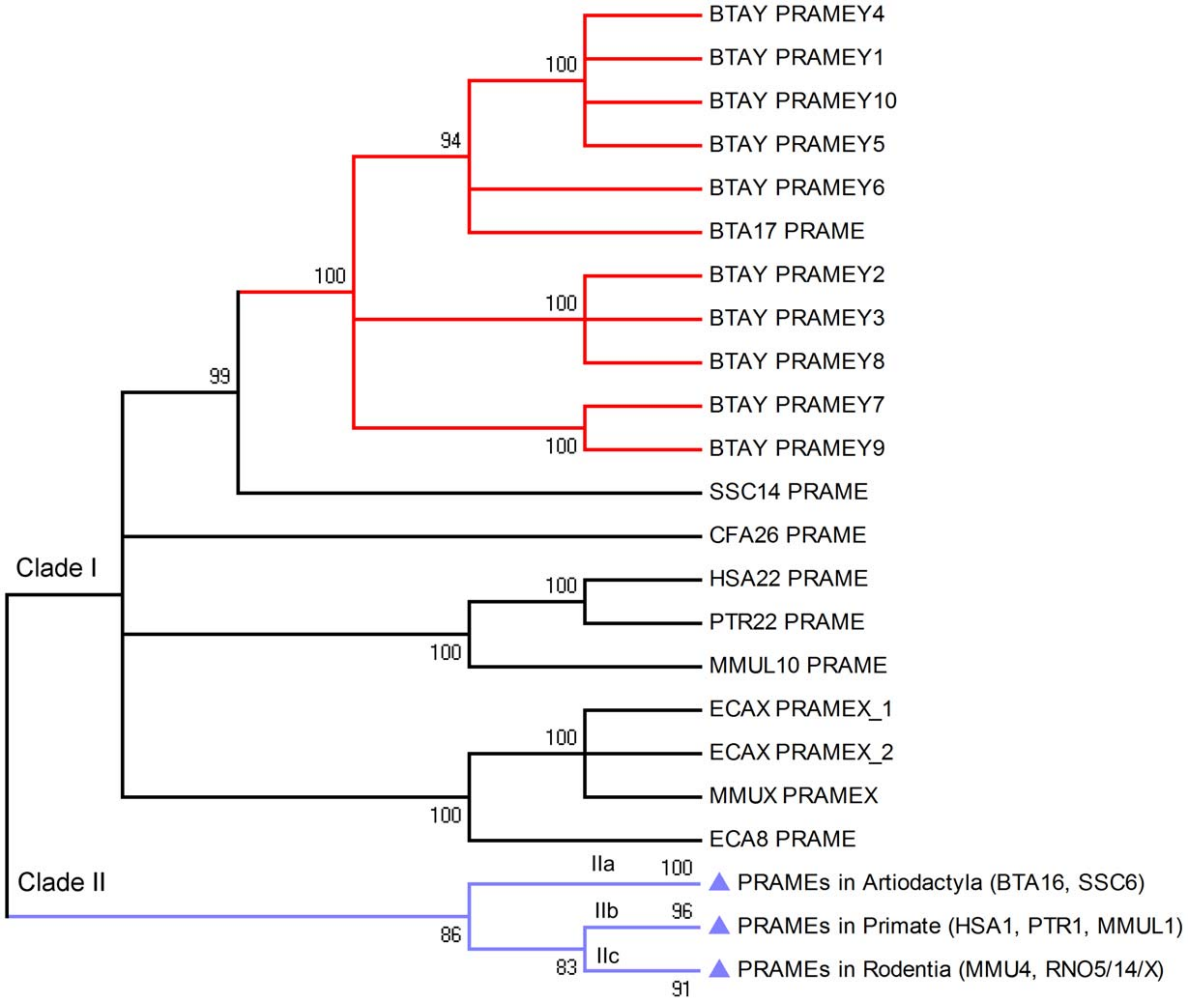
Phylogenetic tree of the *PRAME* gene family. Two major *PRAME/PRAMEY* clades are shown in this tree. The *PRAME* locus on HSA22 and its syntenic orthologs in other species are clustered with the bovine *PRAME* and *PRAMEY* loci in Clade I (branches in red). The orthologs on the X-chrs of horse and mouse are also clustered with Clade I. The *PRAME* orthologs syntenic to HSA1 are clustered in Clade II (branches in light blue), which contains three sub-clusters, IIa (Artiodactyla), IIb (Primates) and IIc (Rodentia). The tree was built based on the ML method and bootstrap values (1000 replicates) are shown above the branches. The branches corresponding to partitions reproduced in less than 80% bootstrap replicates are collapsed.

The coding regions of the retrieved *PRAME* sequences were used to establish phylogenetic trees using Maximum-likelihood (ML), Bayesian-inference (BI) and Neighbor-joining (NJ) methods. All the tree topologies were consistent and contained two major clades (Fig. 4). The first clade (Clade I) included the syntenic orthologs of the BTA17 *PRAME* on human (HSA22), macaque (MMUL10), chimpanzee (PRT22), dog (CFA26), horse (ECA8) and pig (SSC14). Interestingly, all the active bovine *PRAMEY* loci and *PRAME* on BTA17 were clustered on the same branch with a strong bootstrap support value (100%) (Fig. 4). This clade also included the orthologs on the horse and mouse X-chrs (ECAX and MMUX), which have a closer evolutionary distance to Clade I (0.713) than Clade II (0.814) (Maximum-Composite-Likelihood method) [20]. In Clade I, only the *PRAMEY* gene contains multiple copies, whereas the other homologs are all single-copy genes. Since no Y-linked ortholog was identified among the available Y-chrs of the other eutherian mammals, we propose that the bovine *PRAMEY* was derived by a lineage-specific, autosome-to-Y transposition event.

**Figure 5:**
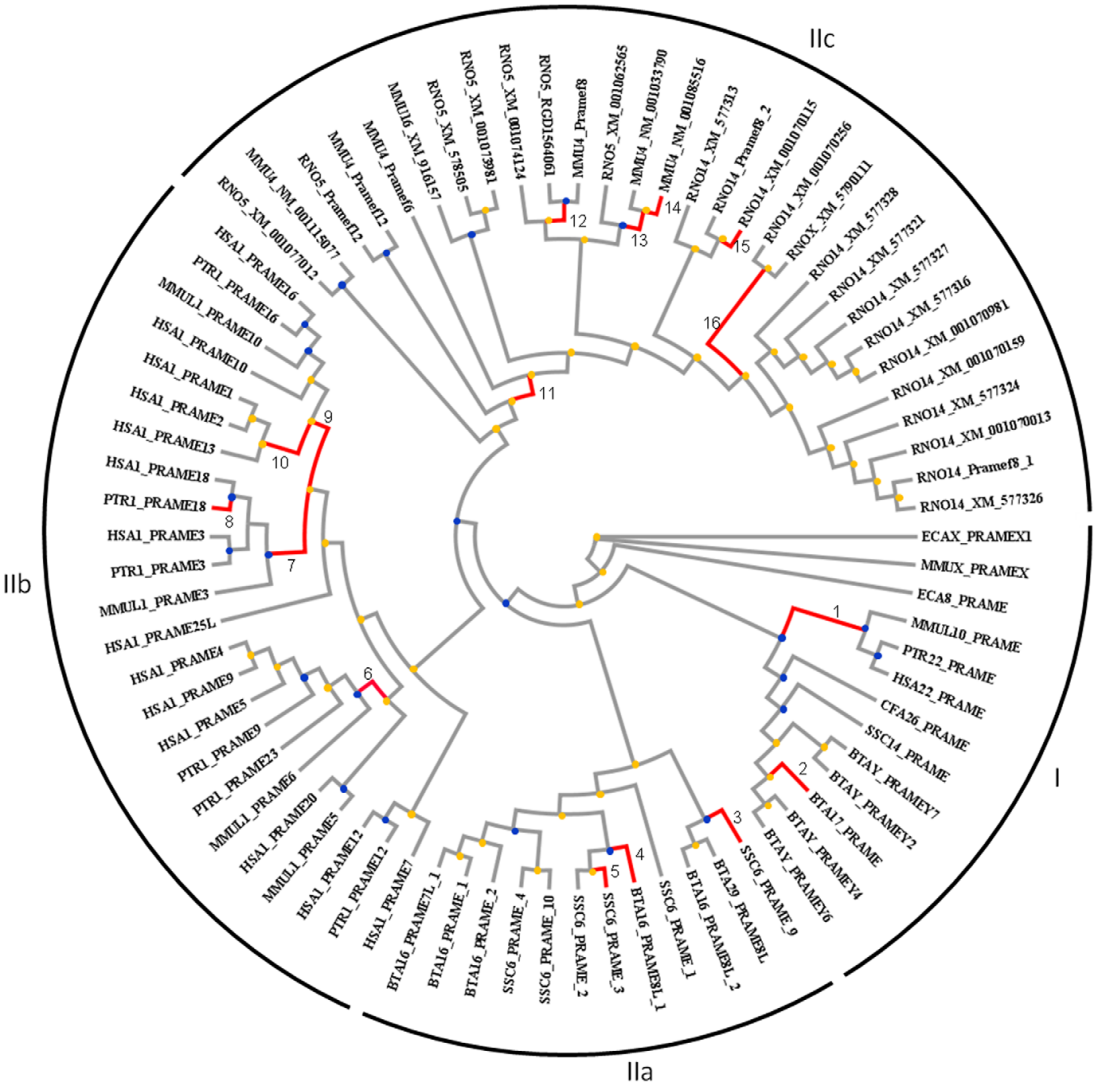
Positive selection on the *PRAME* and *PRAMEY* orthologs. Two branches in Clade I and 14 branches in Clade II are under positive selection (red) based on the branch-site model tests (Model A versus Model A null). The branches under positive selection are numbered and the selected sites along each foreground lineage are detailed in Table S5. The nodes underwent duplication are marked with a yellow circle and speciation with a blue circle.

Clade II included the remaining orthologs with three internal clusters (Fig. 4). The first cluster (IIa) comprised the orthologs in Artiodactyla, including those on BTA16 and SSC6. The second cluster (IIb) included all the orthologs on chr 1 in primates, where the human orthologs were intermingled with chimpanzee and orangutan orthologs as demonstrated previously [13]. The autosomal orthologs in Rodentia constituted the third cluster (IIc) and the mouse and rat orthologs were intermingled within the cluster. The X-linked orthologs in rats were also nested within this cluster. The orthologs in Clade II were all located in a chromosomal region syntenic to HSA1 except for the rat X-orthologs. The *PRAME* gene tree was reconciled with a species tree to reveal potential duplication and speciation events (Fig. 5) [21], [22].

### Selection forces acting on the PRAME genes

**Figure 6:**
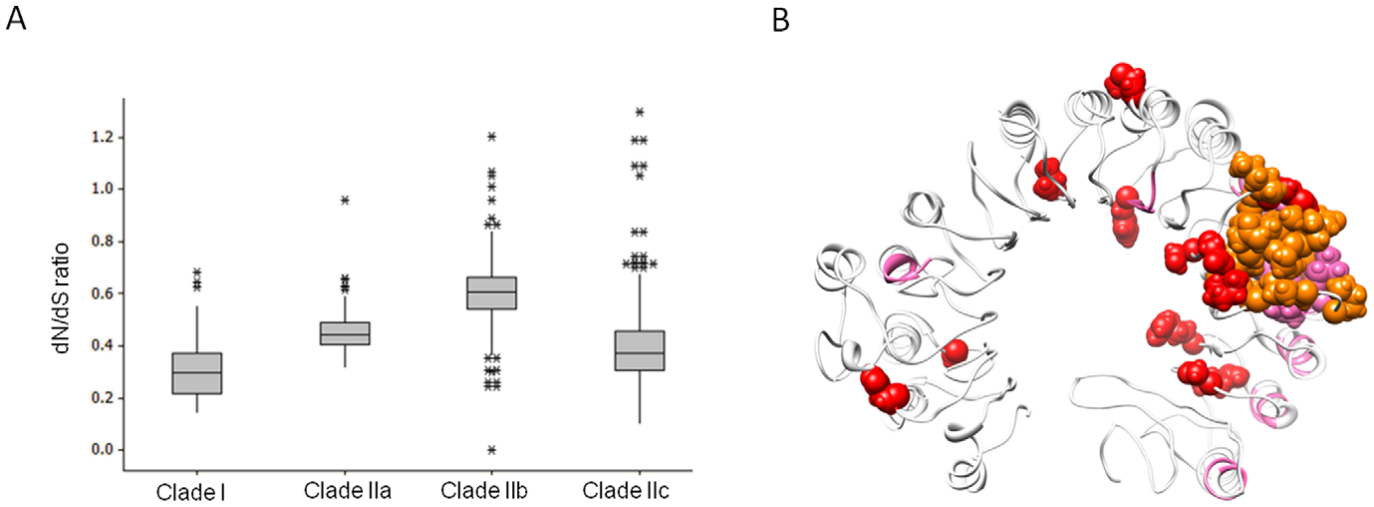
Selective pressures on the *PRAME* family. **A.** The dN/dS ratio distributions in different clades. Clade I has the lowest mean and median dN/dS ratios. The vertical axis represents the dN/dS ratio. The asterisk (*) represents the outliers of the data. **B.** Map of the positively selected sites detected in Clade IIa to the PRAME protein model. The selected sites derived from PAML analyses are mapped to the protein homology model. Eight of the 12 selected sites (red) are clustered in the inner concave region of the protein model. The model was built based on the *PRAME* gene (GenBank acc. no. XM_001256020.1) on BTA16. The predicted DNA binding site is highlighted in orange. The LXXLL motifs are highlighted in pink.

The lineage-specific selection test using the PAML (Phylogenetic Analysis by Maximum Likelihood) package revealed that the dN/dS ratios varied significantly among different lineages (p<0.001, fixed ratio/free ratio branch model) [23]. We applied the branch-site models (model A null/model A) to examine whether any lineage is under positive selection [24]. In Clade I, we observed two branches, leading to the primate homologs and the bovine *PRAME* on BTA17, which were subject to positive selection (Fig. 5). Three positively selected sites were found along these two branches (probability >0.8, Table S5) [24]. We also tested different pairs of site-specific models (see methods) in a dataset containing only the homologs in Clade I (Table S2 and S6) [23], and the results were all negative (p>0.1). It is noteworthy that the homologs in Clade I had a significant lower median dN/dS ratio when compared to the three clusters in Clade II (p<0.001, Fig. 6A). Taken together, these data suggest that Clade I was under stronger functional constraints.

In Clade II, we detected a total of 17 sites from 14 different branches under positive selection (Fig. 5). Four sites and three branches were observed in Artiodactyla (Clade IIa), five sites/five branches in Primate (Clade IIb), and nine sites/six branches in Rodentia (Clade IIc) (Fig. 5). Our findings support a previous report that the primate and rodent *PRAME* homologs were subject to positive selection [13]. In this study, we further examined the potential selected sites in the homologs in Artiodactyla (Clade IIa) using the site-specific models (see methods) and detected eight more positively selected sites (model M8, probability >0.8, Table S6 and S7). Therefore, 12 sites in total were found under positive selection in Clade IIa. We built a PRAME protein homology model using the *PRAME* gene on BTA16 (GenBank acc. no. XM_001256020) as the template, and mapped the positively selected sites on the model (Fig. 5). In contrast to the primate and rodent PRAME, in which the positively selected sites were clustered in the outer surface of the protein [13], the majority (8/12) of the positively selected sites in the bovine PRAME were located in the inner concave region (Fig. 6B). Furthermore, a DNA binding site was predicted in this protein model. This could be important as one of the positively selected sites (329M) and two of the leucine-rich motifs were located in this region. In addition, we also investigated whether or not the bovine paralogs, including the pseudogenes, were subject to gene conversion during evolution using the GENECONV program [25]. The results did not indicate any gene conversion events.

## Discussion

### Lineage-specific amplification of *PRAME*s

*PRAME* is one of the most amplified gene families in mammals and is considered the third largest gene family in the mouse genome [26]. In the present study, we found that the *PRAME* gene family is present only in Eutheria, indicating that this family may have originated *de novo* in the eutherian lineages [27]. The birth-and-death model of gene duplication, instead of concerted evolution, has been suggested to be the major evolutionary mechanism accounting for the expansion of autosomal *PRAME* and the resemblance between each copy [13]. Our analysis revealed that: 1) during eutherian evolution, the expansion of *PRAME* genes was not limited to autosomes, but also occurred in sex chrs; 2) the expansion of *PRAMEs* is lineage-dependent. This conclusion was based upon the finding that the *PRAME* gene was transposed to and amplified on BTAY, but not on the other mammalian Y-chrs; 3) the intra- (cis-) and inter- (trans-) chromosomal duplications occurred during the expansion of the *PRAME* gene family. The cis-duplications occurred mainly for the syntenic *PRAMEs* in Clade II and the bovine *PRAMEYs* in Clade I (Fig.4, Table S2). The rat X-orthologs may be derived from the trans-duplication of the autosomal paralogs on RNO14, but the origin of the mouse X-ortholog is unclear (Fig. 4). It is noteworthy that the *PRAME* genes appear to (cis-) duplicate largely only on those chromosomal regions syntenic to HSA1 in Clade II. In contrast, the orthologs clustered with the *PRAME* gene on HSA22 tend to be maintained as single-copy genes in the respective genome, except for the bovine *PRAMEY* family, which could be a consequence of abundant reorganization and duplication events that occurred during the evolution of the Y-chr [28]. We observed five BACs, each containing two *PRAMEY* loci (Table S1), suggesting that the expansion of *PRAMEY* occurred in tandem on BTAY and gene duplication was the predominant process during the expansion. However, we cannot exclude the possibility that concerted evolution may also have contributed to the similarity between the *PRAMEY* genes because of potential Y-Y gene conversions [29], [30]. The mechanism behind the frequent cis-duplications and limited trans-duplications of the *PRAME* gene family in Eutheria may be related to genomic contexts on each chromosome, including local gene density, repeat density, GC content and recombination rate [31].

### Selective pressures on *PRAME(Y)*

Positive selection tends to increase the frequency of advantageous mutations; negative selection eliminates the deleterious mutations resulting in less genetic variation. A previous study found a large number of positively selected sites in both human and mouse *PRAME* orthologs on HSA1 and MMU4 [13]. In the present study, we found several branches leading to the orthologs in primates, rodents and artiodactyls in Clade II under positive selection (Fig. 5), which supports the previous report [13]. The selection test for the homologs on BTA16 and SSC6 detected 12 sites that were subject to positive selection (Fig. 6B, Table S5 and S7). Unlike the primate [13], the positively selected sites in Artiodactyla were clustered in the inner concave region, suggesting that the functional accommodations of *PRAMEs* are lineage-dependent. The protein structure of the bovine PRAME model (Fig. 6B) is close to the ribonuclease inhibitor (PDB: 1DFJ), which interacts with its substrate through a similar concave region [32]. Thus, the modifications of PRAME in Artiodactyla appear to occur along the regions essential for protein interaction during evolution. Further, the difference in the median dN/dS ratios between Clade I and Clade II (Fig. 6A) suggested differential selection pressures acting on the *PRAME* gene family.

### Origin of *PRAMEY* in cattle

Our recent study [29] in cattle has shown that a gene block containing *ZNF280BY* and *ZNF280AY* was transposed from BTA17 and duplicated on the Y-chr after the transposition. In the present study, we found a *PRAME* on BTA17, which is linked to *ZNF280B*/*ZNF280A* within a 60 kb region (74.30–74.36 Mb). Meanwhile, the same gene order (*ZNF280BY* -*ZNF280AY-PRAMEY)* was observed in two non-overlapping Y-BACs (GenBank acc. no. AC234853.4 and AC233215.5), leading us to hypothesize that the *PRAMEY*s were derived from the transposition of the block on BTA17. Unlike the human *DAZ* and feline *TETY1* and *FLJ36031* genes, in which the translocation was involved in a single autosomal gene, the bovine *ZNF280B-ZNF280A-PRAME* was transposed to the Y-chr as a block. However, the established phylogenetic tree of *PRAME/PRAMEY* in this study was not clear because the BTA17 locus was nested within the *PRAMEY* cluster (Fig. 3), raising an alternative but unlikely hypothesis that the *PRAME* on BTA17 was derived from the loci on BTAY. If we assume a “Y-to-autosome” transposition occurred during evolution, we would expect this gene block to be conserved on the Y-chr of most, if not all, eutherians, but not conserved on autosomes. However, this block is highly conserved on autosomes (Fig. S2) instead of the Y, which apparently conflicts with the alternative hypothesis. Thus, we proposed that the *PRAMEY* genes in cattle were derived from the transposition of the *ZNF280B/ZNF280A/PRAME* on BTA17 and duplicated separately thereafter.

Furthermore, based on the tree topology (Fig. 3), it appears that *PRAMEYs* were clustered into two subgroups and could be derived from two transposition events. However, several observations led us to postulate that *PRAMEY*s were derived from a single transposition of the BTA17 gene block. First, all *PRAMEYs* are highly similar (>86%) and amplify tandemly in a narrow genomic region just like the *PRAME* expansion within 740 kb on HSA1 [13]. Several Y-BACs contain two copies of *PRAMEY*, such as *PRAMEY2* and *PRAMEY3,* which are identical and located in a BAC with a distance of 22 kb. More importantly, *PRAMEY6* and *PRAMEY7,* falling into different subgroups, are also located in one Y-BAC with a distance of 97 kb (Table S1). The narrow distance and high similarity of each copy indicated that the gene duplication is the major evolutionary mechanism of *PRAMEYs* after transposition. Two separate transpositions occurring within a narrow genomic region are implausible. Thus, we propose that the distinct clusters of *PRAMEYs* are the synergic consequence of a higher mutation rate on the non-recombining Y-chr [33] and Y-Y gene conversions [29], [30]. The diversity of the duplicated *PRAMEY* sequences reflects a response of Y-chr to diverse selection pressures.

### Potential roles of *PRAME/PRAMEY*

Several lines of evidence have indicated a close relationship between *PRAME* and tumorigenesis [12], [13], [15], [16], [34]. *PRAME* acts as a ligand-dependent co-repressor in the important retinoic acids receptor (RAR) pathway [12], [34]. When PRAME is absent, the activation of the RAR pathway by retinoids will lead to proliferation arrest, cell differentiation and apoptosis [12]. Conversely, the RAR pathway is inhibited when *PRAME* is abnormally present, resulting in incessant cell proliferation and tumorigenesis.

In addition to tumor development, P*RAME* is implicated in germ cell development. In the mouse, an autosomal *Prame-*like gene, *Oogenesin*, is expressed in oocytes and early cleavage-stage embryos with a role in oogenesis [13], [35], suggesting that the duplicated *PRAME* genes on autosomes are related to rapid cell mitosis. The mouse X-linked *Prame-like 3* (*Pramel3*) is expressed specifically in spermatogonia and may function in early stage of spermatogenesis [36]. Since maintaining and amplifying male fertility factors on the Y-chr may provide selection advantages during evolution [37], the origin and retention of these Y-linked copies are expected to be crucial for spermatogenesis. The exclusive expression of *PRAMEY* (Fig. 1 and 3) in spermatids provides a strong support for this hypothesis. We validated that at least eight of the 10 predicted *PRAMEY* loci are active at the transcription level, and differentially expressed in the testis (Fig. 1B). Future research is needed to investigate the biological meanings behind this differential expression. It is worth noting that the predominant expression of the bovine *PRAMEY2* antisense transcript in spermatid may be essential biologically (Fig. 3E). Our previous works demonstrated that the antisense RNAs of three other Y-related and testis-expressed genes (*ZNF280BY*, *DDX3Y* and *CDYL*) in cattle all appear to be expressed in late stage spermatocytes and/or spermatids, indicating that antisense RNA is crucial in the regulation of bovine spermiogenesis [29], [38], [39].

Recent and extensive duplications of *PRAME* and other CT genes in human are consistently involved in adaptive functions including reproduction and immunity [13], [40]. *PRAME* and neighboring *ZNF280BY/ZNF280AY* on HSA22 are reportedly associated with immune responsiveness [41], [42]. Thus, the *PRAME/PRAMEY* gene family may also participate in auto-immunity to sperm, which is prevented by the blood-testis barrier in normal males [43]. Anti-sperm immunity is considered as one of the causes of infertility in humans [44] and it is thus important to clarify the immunological roles of *PRAME* in male-related functions.

In conclusion, we have identified a lineage-specific *PRAMEY* gene family in bovine, which was derived from the transposition of a gene block, *ZNF280B-ZNF280A-PRAME*, on BTA17, and duplicated afterwards. The expansion of *PRAME* genes occurred not only in Primates and Rodentia, but also in Artiodactyla. The phylogenetic analysis revealed two distinct clades of *PRAME* that evolved under different selection forces. The largely amplified autosomal *PRAME*s are under positive selection, whereas the *PRAMEY*s are under stronger functional constraints. The *PRAMEY* gene family is expected to be important in spermatogenesis. We anticipate that future research on the roles of *PRAME* and *PRAMEY* in the crosstalk between the spermatogenesis and immunoresponse will facilitate understanding of both spermatogenesis and tumor developments.

## Materials and Methods

### RNA extraction and cDNA synthesis

Total RNA was extracted from bovine testicular tissue at 4 days, 20 days, 3–4 months, 8 months, and 2 years of age with Trizol® reagent (Invitrogen, Carlsbad, CA, USA). Equal amounts of total RNA from different ages of testes were pooled and treated with DNase I twice (before and after mRNA purification) (Ambion, Austin, Texas, USA). Messenger RNAs were purified from the pooled total RNA (Oligotex; Qiagen, Valencia, CA, USA). First strand cDNAs were synthesized with random hexamers and oligo-T primers using SuperscriptIII reverse transcriptase (Invitrogen, Carlsbad, CA, USA); blunt-ended double-stranded cDNAs were generated as described [45]. Adaptors [phosphorylated oligonucleotides 1 (5′-CTGAGCGGAATTCGTGAGACC-3′) and 2 (5′-CCAGAGTGCTTAAGGCGAGTCAA-3′)] were attached to cDNAs using T4 polynucleotide kinase (NEB, Ipswich, MA, USA)[46]. Adaptor-ligated cDNA products were used for direct testis cDNA selection.

### Direct testis cDNA selection and sequencing

The entire BTAY DNA was isolated by a micro-dissection approach [47]. The DNA fragments were PCR amplified and labeled with biotin-16-dUTP (Roche, Indianapolis, IN, USA) by nick translation (Roche, Indianapolis, IN, USA). Direct testis cDNA selection was detailed in Yang *et al.* (2011) [29] and Del Mastro and Lovett (1997) [46]. The selected cDNAs were PCR-amplified using the adaptor oligo 1 as the primer. Selection efficiency was assessed by qPCR with Y-linked genes, *SRY* and *DDX3Y*, as positive controls and *β-Actin* and *CDYL* as negative controls. PCR products were cloned using a TOPO-TA cloning kit (Invitrogen, Carlsbad, CA, USA). Randomly selected clones (n = 2208) were grown overnight at 37°C in 2 ml, 96-deep-well culture plates. All clones were dot-blotted on nylon transfer membranes and hybridized with ^32^P-dCTP labeled BTAY fragments and PCR fragments of four genes (*HSFY*, *UBE2D3*, *RPL23A*, and *ZNF280B*) that were highly redundant in our test sequencing result. After dot-blot and elimination of the most likely repetitive clones, 753 clones were selected for sequencing. Plasmid DNA was purified by alkaline lysis (Qiagen, Valencia, CA, USA), and sequenced on an ABI-3730XL DNA analyzer at the Pennsylvania State University Genomics Core Facility.

### RT-PCR

Total RNAs were extracted from nine different tissues (testis, liver, kidney, spleen, cerebellum, adrenal gland, longissimus muscle, lymph node, and spinal cord) of a 2-years old bull and ovarian tissue from a mature cow, then treated with DNase I (Ambion, Austin, TX, USA) and reverse transcribed using Superscript™ III First-Strand Synthesis System (Invitrogen, Carlsbad, CA, USA). RT-PCR was performed in 20 µl containing 10 ng cDNA, 200 µM dNTPs, 1.5 mM MgCl_2_, 2.5 µM of each primer, 1 unit Taq DNA polymerase (Bioline, Taunton, MA, USA). The PCR conditions were: 94°C for 7 min followed by 35 cycles each of 95°C for 40 sec, 55°C–65°C for 40 sec, 72°C for 40 sec, with a final extension at 72°C for 7 min. Products were resolved on 1.5% agarose gels with ethidium bromide in 1× TAE buffer.

### RACE

Total RNAs from bovine testis (5–11 days, 3 months, 8 months and 2 years of age) were used for 5′ and 3′ rapid amplification of cDNA ends (RACE). The RACE experiment was conducted essentially as described in Yang *et al.* [29].

### Short-read sequencing for locus-specific expression

The selected cDNAs were sequenced at the National Center for Genome Resources using an Illumina GAIIx. Library construction and sequence methods were described previously [29]. A total of 6,710,574 high-quality paired end reads of 2×36 bp were generated. These reads were aligned to nine unique *PRAMEY* sequences identified through BlastClust [48] with 100% similarity and 100% coverage as the criteria. For aligning the short-reads, the software GSNAP [49] was used as part of the Alpheus pipeline [50]. Two mismatches were allowed during the alignment step and only the reads that hit the reference uniquely were considered for counting towards locus-specific expression. Since the reads were paired end, only the reads where both ends hit the same reference were considered. These counts were further sub-grouped under two categories: (A) both reads are unique hits with at least one of them being exact match and (B) both reads are unique hits & both are exact matches. The read counts in these two categories were considered a measure of expression pertaining to the specific locus.

### Testis tissue section in situ hybridization (ISH)

The bovine testis was fixed [51], embedded in paraffin and sectioned (4 µm). Sense and antisense RNA probes of *PRAMEY* were selected (Table S4) using G-PROBE (Genetyx Co., Tokyo, Japan) and the 120-bp probes were subjected to *in vitro* transcription to produce digoxigenin (DIG)-labeled cRNA with the AmpliScribe T7-Flash Transcription Kit (Epicentre, Madison, WI, USA). Uniform labeling of DIG-labeling was confirmed using the NBT/BCIP detection system (Roche Diagnostics, Indianapolis, IN, USA). ISH was performed as described previously [38], [39]. Serial tissue sections were used for sense and antisense probe hybridizations. The spermatid-specific gene *Protamine 1* (*PRM1*) served as the positive control, while *LNE120* staining was used as the negative control.

### Strand-specific qPCR

First strand sense and antisense cDNAs were developed with strand-specific reverse transcript primers (Table S3) (SuperScript™ III First-Strand Synthesis System, Invitrogen, Carlsbad, CA, USA) from 5–11 day, 3 month, 8 month and >24-month bovine testis total RNA and used as templates for qPCR with gene specific primer sets (Table S3). All qPCRs were performed in the Power SYBR Green PCR Master Mix (Applied Biosystems, CA, USA) and Applied Biosystems 7500 real-time PCR system following the manufacturer's instructions. Amplification conditions were 2 min at 50°C; 10 min at 95°C; followed by 40 cycles of 20 sec at 95°C, 20 sec at 57°C and 30 sec at 72°C. Cycle threshold acquisition used default parameters with CT values for *PRAMEY* sense/antisense RNAs normalized to 18S rRNA in each sample. RNA samples without a reverse transcript served as the negative control. Each qPCR was conducted in duplicate on three independent RNA samples (biological replicates). Significance was evaluated by one-way ANOVA using SAS (SAS Institute Inc., NC, USA).

### Sequence alignment, gene prediction and phylogenetic tree construction

For the identification of bovine *PRAME* paralogs, we used the two identified transcripts (GenBank acc. no. GU144301 and GU144302) to blast against ∼600 bovine Y-BACs that are available in GenBank to retrieve all potential paralogous regions on BTAY. The redundant regions were removed by detecting the overlaps between Y-BACs using purpose-designed scripts. The paralogs with inferred splicing sites/signals and comparable coding regions were considered as active *PRAMEY* genes; in contrast, the others were pseudogenes due to either frameshift mutations or premature stop codons.

Using the human *PRAME* sequences on HSA22 (GenBank acc. no. NM_206956.1) and HSA1 (GenBank acc. no. NM_023013.1) to blast [48] against the nucleotide databases in NCBI [18], we were able to retrieve the annotated *PRAME* homologs in humans, chimpanzees, orangutans, horses, cats and cattle (e-value <1E^-50^ and coverage >40%, Table S1 and S2). For the swine orthologs, the blast search was against the swine HTGS database as the swine genome sequence was not well annotated. The retrieved porcine BAC sequences were annotated for *PRAME* in this study using Splign [19] and the getorf program in EMBOSS [52]. The redundant porcine paralogs were removed. The identified homologs were used to construct the phylogenetic trees using the ML, BI and NJ methods (substitution model: TrNef + I + G) implemented in the TOPALi program [53]. The alignment gaps were trimmed using Gblocks [54], [55]. The branches with a bootstrap value <80% were collapsed (Fig. 4). We further investigated the duplication and speciation events by reconciling the *PRAME* gene tree with a species tree obtained from NCBI taxonomy database [56] using Notung 2.6 [22] (Fig. 5). No *PRAME* ortholog was identified from the lineages beyond Eutheria, including opossum (6.8x genome coverage), platypus (6x), chicken (6.6x), frog (7.5x) and zebrafish (6.5x).

### Lineage- and site-specific selection test

We conducted a pairwise dN and dS analysis (Dnasp version 5.0) [57] for the orthologs located on the same chromosome across species studied. The sequences with a pairwise dS value of <0.02 were removed, and the resulted 78 sequences were used for lineage-specific positive selection test [23] (Table S1). The median dN/dS ratio was calculated for different clades and compared by the Mann-Whitney test [58]. The 78 sequences were aligned by ClustalW [59] and the gaps were trimmed by Gblocks. The aligned segments included 912 positions of the original 2677 positions. We used the codeml program implemented in PAML package for the selection test. A simple model assuming a single dN/dS ratio for branches was compared with another model assuming free dN/dS ratio for all the branches (branch models). The likelihood ratio test (LRT) indicates that the dN/dS ratios are significantly varied among lineages (p<0.001, 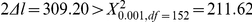). We conducted LRT for each branch using the branch-site models, model A null and model A [24]. The sites under positive selection detected by Bayes Empirical Bayes (BEB) analyses were retrieved when the LRTs were significant.

For the site-specific positive selection test [23], [60], we focused on investigating the Clade I and Clade IIa, which were newly identified in this study. We established two datasets, one with the 12 sequences in Clade I and the other with the 12 sequences in Clade IIa (Table S1). The Clade I dataset included 1290 aligned positions of the original 1677 positions; The Clade IIa dataset included 1065 bases of the original 1902 positions. PAML [23] and HyPhy [60] packages were used to detect the selection. We compared four different model pairs, M0 (one-ratio)/M3 (discrete), M1a (nearly neutral)/M2a (positive selection), M7 (beta)/M8 (beta and ω>1), and M8a (beta and ω_s_ = 1)/M8 in PAML. Three methods, SLAC (Single Likelihood Ancestor Counting), FEL (Fixed Effects Likelihood) and REL (Random Effects Likelihood), implemented in HyPhy (Hypothesis Testing Using Phylogenies) package [60] were also used to detect the positive selection sites (Table S7). The protein model of the *PRAME* gene on BTA16 (GenBank acc. no. XM_001256020) was built by I-TASSER [61].

## Supporting Information

Figure S1 **Motif alignment between the bovine PRAMEY and the human PRAME on HSA22.** The aliphatic sites of LXXLL motifs observed on the human PRAME on HSA22 [16], [12] are conserved in the bovine PRAME(Y). These motif modifications are restricted to the aliphatic group, including the leucine to valine in the third and seventh motifs and leucine to isoleucine in the fourth motif. An exception is that the first leucine in the fifth motif was modified to the non-aliphatic phenylalanine. The colors in the alignment indicated different types of amino acids (White: Aliphatic sites; Red: Acidic sites; Cyan: Basic sites; Purple: Aromatic sites; Yellow: Cystenine). * The aliphatic site positions were annotated based on the PRAME on HSA22.
(TIF)

Figure S2 **Alignment of the** ***ZNF280B*** **/** ***ZNF280A*** **/** ***PRAME*** **gene block across 17 species.** The *ZNF280B*/*ZNF280A*/*PRAME* gene blocks are conserved in the syntenic regions in most mammals except the rodents where the block was rearranged in two different chromosomes (MMU4/10 and RNO5/20). This plot was generated based on the HSA22 assembly (hg19, Feb. 2009). The boxes represent ungapped alignments; the lines represent gaps. This plot was generated using blastz alignment from the UCSC genome browser (http://genome.ucsc.edu/).
(TIF)

Table S1 **A list of BACs containing homologous** ***PRAMEY.***
(DOC)

Table S2 ***PRAME/PRAMEY*** **homologs in the phylogenetic tree.**
(DOC)

Table S3 **Primers for (RT-) PCR and strand-specific qRT-PCR.**
(DOC)

Table S4 **Probes for** ***in situ*** **hybridization.**
(DOC)

Table S5 **Positively selected sites detected from branch-site model tests.**
(DOC)

Table S6 **Site-specific selection tests on the homologs in Clade I and Clade IIa.**
(DOC)

Table S7 **The integrative analysis of positively selected sites in Clade IIa.**
(DOC)
