# Supplementary figures and images for: The Expansion of the PRAME Gene Family in Eutheria
Source: PLoS One. 2011 Feb 10;6(2):e16867. doi: 10.1371/journal.pone.0016867 (PMC3037382; doi:10.1371/journal.pone.0016867)

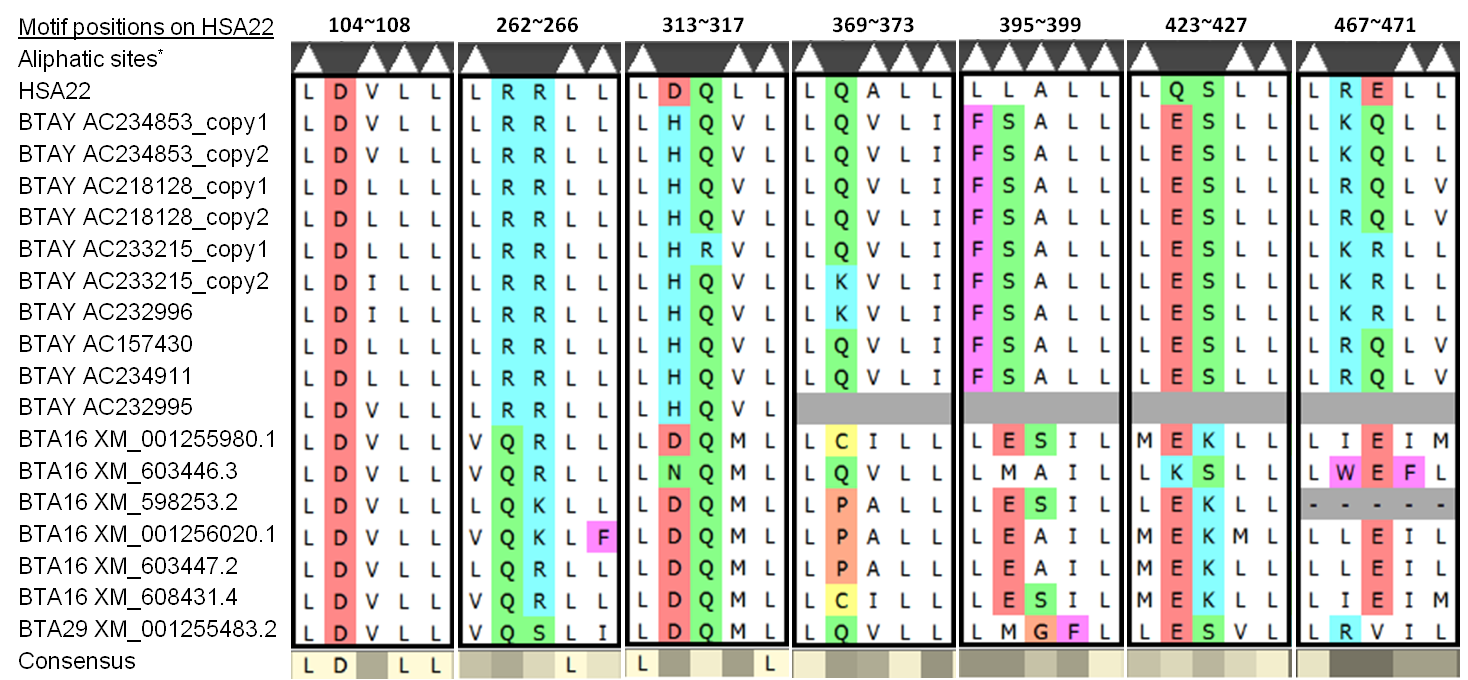

Supplement: Figure S1 — Motif alignment between the bovine PRAMEY and the human PRAME on HSA22. The aliphatic sites of LXXLL motifs observed on the human PRAME on HSA22 [16], [12] are conserved in the bovine PRAME(Y). These motif modifications are restricted to the aliphatic group, including the leucine to valine in the third and seventh motifs and leucine to isoleucine in the fourth motif. An exception is that the first leucine in the fifth motif was modified to the non-aliphatic phenylalanine. The colors in the alignment indicated different types of amino acids (White: Aliphatic sites; Red: Acidic sites; Cyan: Basic sites; Purple: Aromatic sites; Yellow: Cystenine). * The aliphatic site positions were annotated based on the PRAME on HSA22. (TIF) [file pone.0016867.s001.tif]

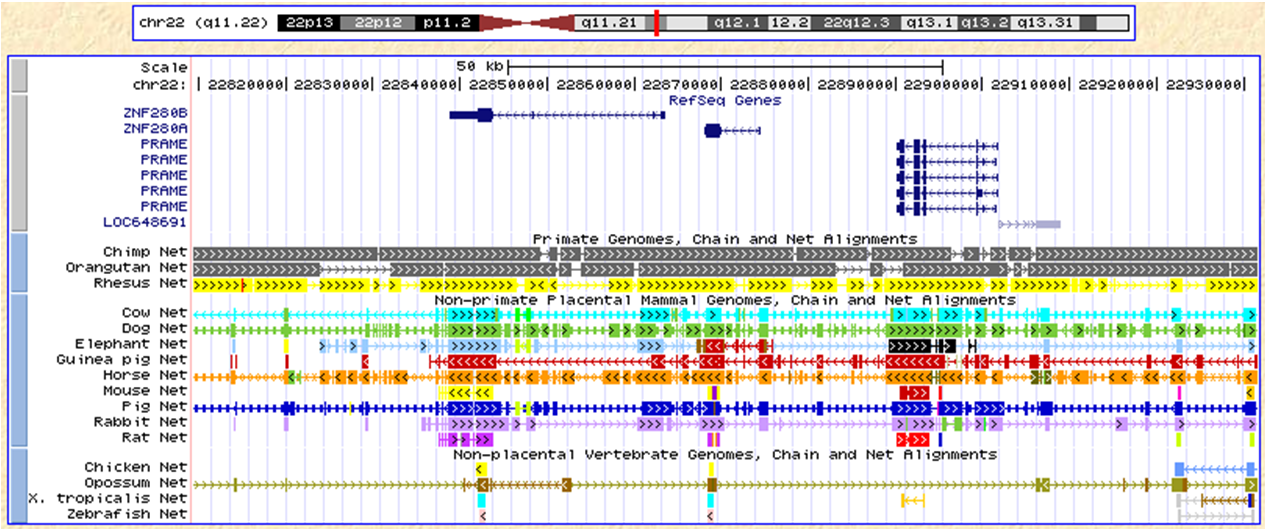

Supplement: Figure S2 — Alignment of the ZNF280B / ZNF280A / PRAME gene block across 17 species. The ZNF280B/ZNF280A/PRAME gene blocks are conserved in the syntenic regions in most mammals except the rodents where the block was rearranged in two different chromosomes (MMU4/10 and RNO5/20). This plot was generated based on the HSA22 assembly (hg19, Feb. 2009). The boxes represent ungapped alignments; the lines represent gaps. This plot was generated using blastz alignment from the UCSC genome browser (http://genome.ucsc.edu/). (TIF) [file pone.0016867.s002.tif]
