# Supplementary material for: The Expansion of the PRAME Gene Family in Eutheria
Source: PLoS One. 2011 Feb 10;6(2):e16867. doi: 10.1371/journal.pone.0016867 (PMC3037382; doi:10.1371/journal.pone.0016867)
Supplement: Table S1 — A list of BACs containing homologous PRAMEY. (DOC) [file pone.0016867.s003.doc]

***Table S1. A list of BACs containing homologous PRAMEYs***

| BAC Accesion NO. | Gene Symbol | Active (A)/ Pesudogenized (P) | Mapping positions | Annotation |
| --- | --- | --- | --- | --- |
| AC234911 | *PRAMEY1* | A | 4642~31956 (unordered segments) | Working Draft |
| AC234853 copy1 | *PRAMEY2* | A | 84805~88744 | Y |
| AC234853 copy2 | *PRAMEY3* | A | 114796~110856 | Y |
| AC218128 copy1 | *PRAMEY4* | A | 99832~95534 | Y |
| AC218128 copy2 | *PRAMEY5* | A | 122570~118264 | Y |
| AC233215 copy1 | *PRAMEY6* | A | 35775~31490 | Y |
| AC233215 copy2 | *PRAMEY7* | A | 137307~132965 | Y |
| AC232995 | *PRAMEY8* | A | 14325~17766 | Working Draft |
| AC232996 copy 1 | *PRAMEY9* | A | 28559-28523 | Working Draft |
| AC157430 | *PRAMEY10* | A | 11676-7259 | Sequencing |
| AC232996 copy2 | - | P | 86662-80509 | Working Draft |
| AC214585 | - | P | 4287-7284 | Working Draft |
| AC215832 | - | P | 14630-17615 | Working Draft |
| AC225835 copy1 | - | P | 114809-117778 | Working Draft |
| AC225835 copy2 | - | P | 41526-44511 | Working Draft |
| AC232754 | - | P | 193691-196054 | Y |
| AC233172 | - | * | 162459-158494 | Y |

*AC233172 overlaps with AC233215
