# Supplementary material for: The Expansion of the PRAME Gene Family in Eutheria
Source: PLoS One. 2011 Feb 10;6(2):e16867. doi: 10.1371/journal.pone.0016867 (PMC3037382; doi:10.1371/journal.pone.0016867)
Supplement: Table S2 — PRAME/PRAMEY homologs in the phylogenetic tree. (DOC) [file pone.0016867.s004.doc]

**Table S2. *PRAME/PRAMEY* homologs in the phylogenetic** tree

| **Gene labels in the tree** | **Species** | **Accession Number** | **Chromosome** | **Test^Δ^** |
| --- | --- | --- | --- | --- |
| HSA1 *PRAME1* | Homo sapiens | NM_023013.1 | 1 | L |
| HSA1 *PRAME2* | Homo sapiens | NM_023014.1 | 1 | L |
| HSA1 *PRAME3* | Homo sapiens | NM_001013692.1 | 1 | L |
| HSA1 *PRAME4* | Homo sapiens | NM_001009611.2 | 1 | L |
| HSA1 *PRAME5* | Homo sapiens | NM_001013407.1 | 1 | L |
| HSA1 *PRAME6* | Homo sapiens | NM_001010889.2 | 1 | N |
| HSA1 *PRAME7* | Homo sapiens | NM_001012277.1 | 1 | L |
| HSA1 *PRAME8* | Homo sapiens | NM_001012276.1 | 1 | N |
| HSA1 *PRAME9* | Homo sapiens | NM_001010890.1 | 1 | L |
| HSA1 *PRAME10* | Homo sapiens | NM_001039361.3 | 1 | L |
| HSA1 *PRAME12* | Homo sapiens | NM_001080830.1 | 1 | L |
| HSA1 *PRAME13* | Homo sapiens | NM_001024661.1 | 1 | L |
| HSA1 *PRAME15* | Homo sapiens | NM_001098376.2 | 1 | N |
| HSA1 *PRAME16* | Homo sapiens | NM_001045480.1 | 1 | L |
| HSA1 *PRAME17* | Homo sapiens | NM_001099851.1 | 1 | N |
| HSA1 *PRAME18* | Homo sapiens | NM_001099850.1 | 1 | L |
| HSA1 *PRAME19* | Homo sapiens | NM_001099790.1 | 1 | N |
| HSA1 *PRAME20* | Homo sapiens | NM_001099852.1 | 1 | L |
| HSA1 *PRAME21* | Homo sapiens | NM_001100114.1 | 1 | N |
| HSA1 *PRAME22* | Homo sapiens | NM_001100631.1 | 1 | N |
| HSA1 *PRAME23* | Homo sapiens | XM_001130065.3 | 1 | N |
| HSA1 *PRAME25L* | Homo sapiens | XM_497653.4 | 1 | L |
| HSA22 *PRAME* | Homo sapiens | NM_206956.1 | 22 | LS |
| PTR1 *PRAME12* | Pan troglodytes | XM_001139055.1 | 1 | L |
| PTR1 *PRAME23* | Pan troglodytes | XM_525206.2 | 1 | L |
| PTR1 *PRAME9* | Pan troglodytes | XM_001153199.1 | 1 | L |
| PTR1 *PRAME18* | Pan troglodytes | XM_001154638.1 | 1 | L |
| PTR1 *PRAME3* | Pan troglodytes | XM_525207.2 | 1 | L |
| PTR1 *PRAME16* | Pan troglodytes | XM_001147639.1 | 1 | L |
| PTR22 *PRAME* | Pan troglodytes | XM_001148586.1 | 22 | LS |
| MMUL1 *PRAME6* | Macaca mulatta | XM_001118879.1 | 1 | L |
| MMUL1 *PRAME5* | Macaca mulatta | XM_001106867.1 | 1 | L |
| MMUL1 *PRAME3* | Macaca mulatta | XM_001106795.1 | 1 | L |
| MMUL1 *PRAME10* | Macaca mulatta | XM_001106552.1 | 1 | L |
| MMUL10 *PRAME* | Macaca mulatta | XM_001091109.1 | 10 | LS |
| SSC6 *PRAME_*1 | Sus scrofa | FP102722.2 (125652~121760) | 6 | LS |
| SSC6 *PRAME_*2 | Sus scrofa | FP102722.2 (103434~107383) | 6 | LS |
| SSC6 *PRAME_*3 | Sus scrofa | FP102722.2 (140819~138188) | 6 | LS |
| SSC6 *PRAME_*4 | Sus scrofa | FP102722.2 (89830~87260) | 6 | LS |
| SSC6 *PRAME_*5 | Sus scrofa | FP102722.2 (83236~80611) | 6 | N |
| SSC6 *PRAME_*6 | Sus scrofa | FP103101.3(174307~171181) | 6 | N |
| SSC6 *PRAME_*7 | Sus scrofa | FP103101.3 (188225~191309) | 6 | N |
| SSC6 *PRAME_*8 | Sus scrofa | FP103101.3 (195313~197944) | 6 | N |
| SSC6 *PRAME_*9 | Sus scrofa | FP103101.3 (209252~199050) | 6 | LS |
| SSC6 *PRAME_*10 | Sus scrofa | FP103101.3 (136519~133391) | 6 | LS |
| SSC14 *PRAME* | Sus scrofa | NW_001885456.1(1417788~1414476) | 14 | LS |
| CFA26 *PRAME* | Canis familiaris | XM_534747.2 | 26 | LS |
| ECA8 *PRAME* | Equus caballus | XM_001488376.2 | 8 | LS |
| BTA17 *PRAME* | Bos taurus | XR_082974.1 | 17 | N |
| BTAY *PRAMEY1** | Bos taurus | GU144301 (AC234911.1 ) | Y | DN |
| BTAY *PRAMEY2** | Bos taurus | GU144302 (AC234853.4) | Y | DLS |
| BTAY *PRAMEY3** | Bos taurus | AC234853.4 | Y | N |
| BTAY *PRAMEY4** | Bos taurus | AC218128.5 | Y | DLS |
| BTAY *PRAMEY5** | Bos taurus | AC218128.5 | Y | DN |
| BTAY *PRAMEY6** | Bos taurus | AC233215.5 | Y | DLS |
| BTAY *PRAMEY7** | Bos taurus | AC233215.5 | Y | DLS |
| BTAY *PRAMEY8** | Bos taurus | AC232995.3 | Y | DN |
| BTAY *PRAMEY9** | Bos taurus | AC232996.5 | Y | N |
| BTAY *PRAMEY10** | Bos taurus | AC157430.2 | Y | DN |
| BTA16 *PRAME8L_1* | Bos taurus | XM_001255980.1 | 16 | LS |
| BTA16 *PRAME7L_1* | Bos taurus | XM_598253.2 | 16 | LS |
| BTA16 *PRAME8L_2* | Bos taurus | XM_603446.3 | 16 | LS |
| BTA16 *PRAME_1* | Bos taurus | XM_001256020.1 | 16 | LS |
| BTA16 *PRAME_2* | Bos taurus | XM_603447.2 | 16 | LS |
| BTA16 *PRAME7L_2* | Bos taurus | XM_608126.2 | 16 | N |
| BTA29 *PRAME8L* | Bos taurus | XM_001255483.2 | 29 | LS |
| ECAX _PRAMEX1 | Equus caballus | XM_001914824.1(NW_001877045.1) | X | LS |
| ECAX _PRAMEX2 | Equus caballus | XM_001915010.1(NW_001877046.1) | X | N |
| MMUX *PRAMEX* | Mus musculus | NM_029459.2 | X | LS |
| MMU4 _NM_001115077 | Mus musculus | NM_001115077.1 | 4 | L |
| MMU4 *Pramef6* | Mus musculus | NM_001085414.2 | 4 | L |
| MMU4_ NM_001085516 | Mus musculus | NM_001085516.1 | 4 | L |
| MMU4_NM_001033790 | Mus musculus | NM_001033790.3 | 4 | L |
| MMU4 *Pramef12* | Mus musculus | NM_029948.2 | 4 | L |
| MMU4 *Pramef8* | Mus musculus | NM_172877.2 | 4 | L |
| MMU16_XM_916157 | Mus musculus | XM_916157.1 | 16 | L |
| RNOX_XM_579011 | Rattus norvegicus | XM_579011.2 | X | L |
| RNOX_XM_001066642 | Rattus norvegicus | XM_001066642.1 | X | N |
| RNO14_XM_001070981 | Rattus norvegicus | XM_001070981.1 | 14 | L |
| RNO14_XM_001070256 | Rattus norvegicus | XM_001070256.1 | 14 | L |
| RNO14_XM_001070159 | Rattus norvegicus | XM_001070159.1 | 14 | L |
| RNO14_XM_001070115 | Rattus norvegicus | XM_001070115.1 | 14 | L |
| RNO14_XM_001070013 | Rattus norvegicus | XM_001070013.1 | 14 | L |
| RNO14_XM_577326 | Rattus norvegicus | XM_577326.2 | 14 | L |
| RNO14 *Pramef8_1* | Rattus norvegicus | XR_006313.1 | 14 | L |
| RNO14 *Pramef9* | Rattus norvegicus | XM_001065244.1 | 14 | N |
| RNO14_XM_577320 | Rattus norvegicus | XM_577320.2 | 14 | N |
| RNO14_XM_577319 | Rattus norvegicus | XM_577319.2 | 14 | N |
| RNO14_XM_577318 | Rattus norvegicus | XM_577318.2 | 14 | N |
| RNO14_XM_577313 | Rattus norvegicus | XM_577313.2 | 14 | L |
| RNO14 *Pramef8_2* | Rattus norvegicus | XM_577363.1 | 14 | L |
| RNO14_XM_577328 | Rattus norvegicus | XM_577328.1 | 14 | L |
| RNO14_XM_577327 | Rattus norvegicus | XM_577327.1 | 14 | L |
| RNO14_XM_577324 | Rattus norvegicus | XM_577324.1 | 14 | L |
| RNO14_XM_577321 | Rattus norvegicus | XM_577321.1 | 14 | L |
| RNO14_XM_577316 | Rattus norvegicus | XM_577316.1 | 14 | L |
| RNO14_XM_577312 | Rattus norvegicus | XM_577312.1 | 14 | N |
| RNO5_XM_001074124 | Rattus norvegicus | XM_001074124.1 | 5 | L |
| RNO5_XM_001073981 | Rattus norvegicus | XM_001073981.1 | 5 | L |
| RNO5_XM_001073804 | Rattus norvegicus | XM_001073804.1 | 5 | N |
| RNO5_RGD1564061 | Rattus norvegicus | NM_001135782.1 | 5 | L |
| RNO5_XM_001062565 | Rattus norvegicus | XM_001062565.1 | 5 | L |
| RNO5 *Pramef12* | Rattus norvegicus | XM_001077034.1 | 5 | L |
| RNO5_XM_001077012 | Rattus norvegicus | XM_001077012.1 | 5 | L |
| RNO5_XM_578508 | Rattus norvegicus | XM_578508.2 | 5 | N |
| RNO5_XM_578506 | Rattus norvegicus | XM_578506.2 | 5 | N |
| RNO5_XM_578505 | Rattus norvegicus | XM_578505.2 | 5 | L |
| RNO5_XM_578482 | Rattus norvegicus | XM_578482.1 | 5 | N |

* Predicted in this study

Δ The sequences with pairwise dS value smaller than 0.02 are not used for PAML analysis (N). D: Aligned with deep sequence reads; L: lineage-specific test and branch-site model tests; S: site-specific test.
