# Supplementary material for: The Expansion of the PRAME Gene Family in Eutheria
Source: PLoS One. 2011 Feb 10;6(2):e16867. doi: 10.1371/journal.pone.0016867 (PMC3037382; doi:10.1371/journal.pone.0016867)
Supplement: Table S3 — Primers for (RT-) PCR and strand-specific qRT-PCR. (DOC) [file pone.0016867.s005.doc]

**Table S3.** **Primers for (RT-) PCR and strand-specific qRT-PCR**

| Primer name | Sequence (5’- 3’) | Annealing temp (℃) | Purpose |
| --- | --- | --- | --- |
| PRAMEY_ASRT | AACTGCATCTGGAAGCTGGC |  | Antisense strand reverse transcript primer |
| PRAMEY_SRT | GGCAATTGGTGATCGACAGG |  | Sense strand reverse transcript primer |
| PRAMEYqF | TGGCCACGCTGAGCAGGTTC | 57 | Strand specific quantitative PCR forward primer |
| PRAMEYqR | ATATGTGGATGCGAGACAGC |  | Strand specific quantitative PCR reverse primer |
| PRAMERTF | CGGGAGGAGCACTGCGTTGG | 57 | BTA17 copy RT-PCR forward primer for expression pattern |
| PRAMERTR | AGGACCTGCAGGGGCTCCAG |  | BTA17 copy RT-PCR reverse primer for expression pattern |
| PRAME1310F | GACCTGGAGGAGTGCCGCAT | 64 | Amplification of XR_082974.1 |
| PRAME1659R | TGGGCTCGGGGTCATAGAAG | 64 | Amplification of XR_082974.1 |
| PRAMEYRTF | AACTGCGTCAACCAGCTCAG |  | AC234853 copy RT-PCR forward primer for expression pattern |
| PRAMEYRTR | AATCAGGCAATTGGTGATCG |  | AC234853 copy RT-PCR reverse primer for expression pattern |
| PRAMEYB1F | GGATTTGCACCTCCCCAGGG | 60 | AC 234853 copy RT-PCR forward primer to amplify 5' part |
| PRAMEYB1R | AATCAGGCAATTGGTGATCG |  | AC 234853 copy RT-PCR reverse primer to amplify 5' part |
| PRAMEYB2F | AACTGCGTCAACCAGCTCAG | 60 | AC 234853 copy RT-PCR forward primer to amplify 3' part |
| PRAMEYB2R | TGTGTAGGAACATATCTCTC |  | AC 234853 copy RT-PCR reverse primer to amplify 3' part |
| PRAMEYA1F | AATTGTATTTGTGGGCTTCG | 60 | AC234911 copy RT-PCR forward primer to amplify 5' part |
| PRAMEYA1R | GTTCTGCAGCAGGCTCTGGC |  | AC234911 copy RT-PCR reverse primer to amplify 5' part |
| PRAMEYA2F | GAGTAGGTGCTTCAGGATGG | 58 | AC234911 copy RT-PCR forward primer to amplify middle part |
| PRAMEYA2R | AATCAGGCAGTTGCTGATGC |  | AC234911 copy RT-PCR reverse primer to amplify middle part |
| PRAMEYA3F | CACTGCGTCCGCCAGCTCAC | 60 | AC234911 copy RT-PCR forward primer to amplify 3' part |
| PRAMEYA3R | CAACCTCCCAAGCCCAAAAC |  | AC234911 copy RT-PCR reverse primer to amplify 3' part |
| 18SrRNAqF | CACGGACAGGATTGACAGATTG | 57 | Strand specific quantitative PCR internal control Forward primer |
| 18SrRNAqR | CAAATCGCTCCACCAACTAAGA |  | Strand specific quantitative PCR internal control reverse primer |
| 18SrRNART | GCCTCACTAAACCATCCAATC |  | Reverse transcript primer |
