# Supplementary material for: The Expansion of the PRAME Gene Family in Eutheria
Source: PLoS One. 2011 Feb 10;6(2):e16867. doi: 10.1371/journal.pone.0016867 (PMC3037382; doi:10.1371/journal.pone.0016867)
Supplement: Table S4 — Probes for in situ hybridization. (DOC) [file pone.0016867.s006.doc]

**Table S4.** **Probes for *in situ* hybridization**

| cRNA probe name | Sequence (5’- 3’) |
| --- | --- |
| PRAMEY sense probe (For detection of antisense RNA) | TGGCCACGCTGAGCAGGTTCCTGCCGCACCTGGGCCGGATGGGCAACCTGCGCCGGCTGCTGCTGTCTCGCATCCACATATTGCCACATACCACCCCGGACCAGGAGAACTGCGTCAACC |
| PRAMEY antisense probe (For detection of sense RNA) | GGTTGACGCAGTTCTCCTGGTCCGGGGTGGTATGTGGCAATATGTGGATGCGAGACAGCAGCAGCCGGCGCAGGTTGCCCATCCGGCCCAGGTGCGGCAGGAACCTGCTCAGCGTGGCCA |
| PRM1 (Positive control) | GGCUGGUGACCUUUCAGGACAGGAGUGCGGUGGUCUUGCUACUGUGUGGUUACUGUCUUGUACACCUUAUGACGGUGUAGCGACGGCAGCACACUCUCCUCCUGCGCCUCCGACCAGAGC |
| LNE120 (Negative control) | UGCCUGCAAAGAUGAGGAGGGAUUGCAGCGUGUUUUUAAUGAGGUCAUCACGGGAUCCCAUGUGCGUGACGGACAUCGGGAAACGCCAAAGGAGAUUAUGUACCGAGGAAGAAUGUCGCU |
