# Supplementary material for: The Expansion of the PRAME Gene Family in Eutheria
Source: PLoS One. 2011 Feb 10;6(2):e16867. doi: 10.1371/journal.pone.0016867 (PMC3037382; doi:10.1371/journal.pone.0016867)
Supplement: Table S5 — Positively selected sites detected from branch-site model tests. (DOC) [file pone.0016867.s007.doc]

**Table S**5. Positively selected sites detected from branch-site model tests

| Branch | Clade | Model | ℓ | 2Δℓ | df | p-value | adjusted p-value^a^ | Selected Sites^b^ |
| --- | --- | --- | --- | --- | --- | --- | --- | --- |
| 1 | I | A | -28579.545037 | 16.10394 | 1 | 5.99591E-05 | 2.99796E-05 | 22S |
| 1 | I | A null | -28587.597008 |  |  |  |  |  |
| 2 | I | A | -28577.520125 | 17.39372 | 1 | 3.03828E-05 | 1.51914E-05 | 261N,412S |
| 2 | I | A null | -28586.216984 |  |  |  |  |  |
| 3 | IIa | A | -28584.719606 | 5.774652 | 1 | 0.01625894 | 0.00812947 | 325E,352S |
| 3 | IIa | A null | -28587.606932 |  |  |  |  |  |
| 4 | IIa | A | -28584.700497 | 5.557438 | 1 | 0.018402327 | 0.009201164 | 42I |
| 4 | IIa | A null | -28587.479216 |  |  |  |  |  |
| 5 | IIa | A | -28585.483816 | 4.395984 | 1 | 0.036023672 | 0.018011836 | 321M |
| 5 | IIa | A null | -28587.681808 |  |  |  |  |  |
| 6 | IIb | A | -28586.195413 | 2.956624 | 1 | 0.085526371 | 0.042763185 | n.a. |
| 6 | IIb | A null | -28587.673725 |  |  |  |  |  |
| 7 | IIb | A | -28583.801907 | 6.67422 | 1 | 0.009781732 | 0.004890866 | 407Q |
| 7 | IIb | A null | -28587.139017 |  |  |  |  |  |
| 8 | IIb | A | -28583.468410 | 6.873632 | 1 | 0.008747669 | 0.004373835 | 70K |
| 8 | IIb | A null | -28586.905226 |  |  |  |  |  |
| 9 | IIb | A | -28583.069225 | 4.045548 | 1 | 0.044288003 | 0.022144002 | 350Q,418I |
| 9 | IIb | A null | -28585.091999 |  |  |  |  |  |
| 10 | IIb | A | -28584.163580 | 3.087 | 1 | 0.078920204 | 0.039460102 | 99A |
| 10 | IIb | A null | -28585.707080 |  |  |  |  |  |
| 11 | IIc | A | -28580.547564 | 13.59473 | 1 | 0.000226822 | 0.000113411 | 74Q,309L |
| 11 | IIc | A null | -28587.344927 |  |  |  |  |  |
| 12 | IIc | A | -28586.274628 | 2.81375 | 1 | 0.093459696 | 0.046729848 | 374E |
| 12 | IIc | A null | -28587.681503 |  |  |  |  |  |
| 13 | IIc | A | -28586.130072 | 3.077286 | 1 | 0.079392925 | 0.039696462 | n.a. |
| 13 | IIc | A null | -28587.668715 |  |  |  |  |  |
| 14 | IIc | A | -28586.311340 | 2.740936 | 1 | 0.097807491 | 0.048903746 | n.a. |
| 14 | IIc | A null | -28587.681808 |  |  |  |  |  |
| 15 | IIc | A | -28580.737873 | 7.889712 | 1 | 0.004971678 | 0.002485839 | 74M,119L,121V,123D,131D |
| 15 | IIc | A null | -28584.682729 |  |  |  |  |  |
| 16 | IIc | A | -28584.109264 | 6.445962 | 1 | 0.011120482 | 0.005560241 | 374E |
| 16 | IIc | A null | -28587.332245 |  |  |  |  |  |

a. The probability is adjusted by dividing the inferred LRT probability by 2 [23].

b. Only the sites with posterior probability > 0.8 under Bayes Empirical Bayes (BEB) analysis are shown in this table. The amino acids refer to the *PRAME* sequence on human chromosome 22 (NM_206956.1)
