# Supplementary material for: The Expansion of the PRAME Gene Family in Eutheria
Source: PLoS One. 2011 Feb 10;6(2):e16867. doi: 10.1371/journal.pone.0016867 (PMC3037382; doi:10.1371/journal.pone.0016867)
Supplement: Table S6 — Site-specific selection tests on the homologs in Clade I and Clade IIa. (DOC) [file pone.0016867.s008.doc]

**Table S6. Site-specific selection tests on the homologs in Clade I and Clade IIa**

|  | **Parameter** | **M0** | **M3^b^** | **M1a** | **M2a^b^** | **M7** | **M8a** | **M8^b^** |
| --- | --- | --- | --- | --- | --- | --- | --- | --- |
| Clade I | -ℓ | -9191.703 | -9081.872 | -9123.991 | -9123.991 | -9085.397 | -9085.545 | -9084.739 |
|  | 2Δℓ | 219.662 |  | 0 |  | 1.316 | 1.612 |  |
|  | p-value | <0.0001 |  | 1 |  | 0.518 | 0.102 ^c^ |  |
|  | df | 4 |  | 2 | none | 2 | 1 | none |
|  | Sites with dN/dS ratio > 1^a^ | n.a. | none | n.a. | none | n.a. | n.a. | none |
| Clade IIa | -ℓ | -7081.539 | -6953.777 | -6966.611 | -6956.987 | -6967.962 | -6965.052 | -6954.536 |
|  | 2Δℓ | 127.762 |  | 19.248 |  | 26.852 | 21.032 |  |
|  | p-value | <0.0001 |  | <0.0001 |  | <0.0001 | <0.0001^c^ |  |
|  | df | 4 |  | 2 |  | 2 | 1 |  |
|  | Sites with dN/dS ratio > 1^a^ | n.a. | none | n.a. | 43Q, 226V, 238S, 261M, **336S**, **443S** | n.a. | n.a. | 43Q, 226V, **261M**, 276F, **336S**, 422R, **443S**, 446R |

a. Only the sites with posterior probability > 0.8 under Bayes Empirical Bayes (BEB) analysis are shown in this table.

b. The sites with posterior probability > 0.9 are shown in bold characters. The amino acid refer to the *PRAME* sequence (XM_001256020.1) on BTA16.

c. The probability is adjusted by dividing the inferred LRT probability by 2 [23].
