# Supplementary material for: The Expansion of the PRAME Gene Family in Eutheria
Source: PLoS One. 2011 Feb 10;6(2):e16867. doi: 10.1371/journal.pone.0016867 (PMC3037382; doi:10.1371/journal.pone.0016867)
Supplement: Table S7 — The integrative analysis of positively selected sites in Clade IIa. (DOC) [file pone.0016867.s009.doc]

***Table S7. The integrative analysis of positively selected sites in Clade IIa.***

| PRAME^a^ | Residue | Posterior Probability^b^ | SLAC p-value | FEL p-value | REL Bayes Factor |
| --- | --- | --- | --- | --- | --- |
| 43 | Q | 0.875 | - | - | - |
| 226 | V | 0.875 | 0.142 | 0.015 | 138.093 |
| 261 | M | 0.903 | 0.111 | 0.077 | 79.999 |
| 276 | F | 0.815 | - | - | - |
| 336 | S | 0.937 | 0.286 | 0.192 | 60.491 |
| 422 | R | 0.836 | - | - | - |
| 443 | S | 0.977 | - | - | - |
| 446 | R | 0.829 | 0.076 | 0.006 | 117.530 |

a. The positions are annotated based on a *PRAME* (XM_001256020.1) on BTA16.

b. The sites with posterior probability > 0.8 from the PAML model 8 analysis are reported.
